# Sometimes Mother Nature only whispers

**DOI:** 10.1107/S2052252525009030

**Published:** 2025-10-27

**Authors:** Sven Lidin

**Affiliations:** a Division of Polymer Materials Chemistry Lund University Box 124 SE-221 00Lund Sweden

**Keywords:** iron(II) pyrophosphate, phase transitions, incommensurately modulated structures, twinning

## Abstract

Polyphasic Fe_2_P_2_O_7_ is a prime example of how the temperature-dependent changes in structure shed light on the magnitudes of local interactions. State-of-the-art methods and equipment are prerequisites for the analysis of these, often subtle, effects.

At a phase transition, a material changes structure and properties. The most fundamental phase transitions are obvious to the eye; the melting of ice or the boiling of water. The change of aggregation state is obvious and most of the accompanying changes in properties are unsurprising, even though the change in density on the solidification of water to ice requires a closer look at the structure of the solid. This is where crystallography comes in.

When a phase transition takes place between two solid phases, the need for a structural understanding is more pronounced. The differences between the properties of the solid phases may be extreme, as between diamond and graphite, and it is clear that the structural change must be equally profound, but sometimes it is quite subtle, and that is the case for Fe_2_P_2_O_7_. This does not make the changes less important or less interesting.

Iron(II) pyrophosphate has a structure close to that of the mineral thortveitite – the only mineral that contains scandium as main constituent – but as with many other thortveitite-like structures, it’s a complicated story.

That Fe_2_P_2_O_7_ undergoes multiple, temperature-driven phase transitions has been known for some time, but relation between and the structures of these phases have been under dispute. The problem has been twinning and incommensurability, and the solution, as so often, has turned out to be better data from better equipment, and the identification of an incommensurately modulated structure is at the very heart of the matter.

In the current issue of *IUCrJ*, Berthold Stöger and coworkers show that the four phases, β, α_3_, α_2_ and α_1_, are all related by reversible phase transitions and the structure of each phase is carefully determined (Stöger *et al.*, 2025 ▸).

The high-temperature modification, the β phase, is isostructural with thortveitite, and belongs to the monoclinic space group *C*2/*m*. All the α phases are triclinic. Twinning is rife. It all seems a bit excessive.

What is going on here, and why?

The action is easiest to follow from the perspective of the pyrophosphate group (see Fig. 1 ▸). This is simply two PO_4_ tetrahedra sharing a common oxygen atom, and in the β phase, which is stable from about 190°C to above 1000°C, the P—O—P angle is 180°. Or at least that is what appears to be the case, but the anisotropic displacement parameter is showing a pronounced elongation in a direction perpendicular to the P—O—P axis, indicating that the bridging oxygen may well be dynamically disordered over two positions off-axis.

In the second high-temperature phase, α_3_, stable up to 190°C, the situation is very similar, but the symmetry is clearly lowered to triclinic (*C*1, to make comparisons more straightforward) indicating that the disorder is here static rather than dynamic.

In the room-temperature α_2_ phase, the off-axis bonds start to develop long-distance ordering, giving rise to an incommensurately modulated structure and, finally, in the fully ordered α_1_ phase, the modulation locks into a commensurate value.

Concurrently, the coordination around one of the two independent Fe(II) ions changes from 6-coordinate to 5-coordinate as the pyrophosphate group exhibits increasing deviation from the linear P—O—P arrangement. As always, there is a question of what is the cart and what is the horse in this arrangement. There is probably cooperativity of the two effects.

Why is this important?

Crystallography is all about understanding structure. Conflicting reports in the literature indicate that there is indeed something that we do not understand. Given the difference in scattering power between Fe and O, it is easy to make the assumption that careful modelling of Fe is more important than doing the same job for O.

Nothing could be further from the truth. Modelling Fe carefully is more important in terms of fitting the model to the data, but that is not what crystallography is about. Crystallography is all about understanding structure.

This is not an unusual situation in itself, but it is still a little rare to be able to resolve complex extra ordering that is almost exclusively manifested in the motion of a small number of light atoms in the structure. It is common that hydrogen-bonding patterns break the symmetry of a simpler structure, and the effects are seen mostly as slight deviations in the positions of the atoms affected by the hydrogen bonding, rather than any signal from the hydrogen atoms themselves. Studies using other radiation sources (for hydrogen, typically neutrons applied to a deuterated sample) may yield data better suited to resolve the issue, but this also comes with its own, well known challenges.

Interestingly, this is but one example from four known systems of thortveitite structures that all exhibit multiple phase transitions where one of the phases is an intermediate, incommensurately modulated phase. Similar behaviour is found in Cr_2_P_2_O_7_, Zn_2_P_2_O_7_ and Zn_2_As_2_O_7_.

This study is also important because it shows how far we have come in terms of data quality from in-house equipment. Powerful sources and sensitive detectors allow the use of very small samples that can circumvent the twinning problem that is always present when temperature-driven phase transitions lead to symmetry lowering. In-house measurements never achieve the signal-to-noise ratio available at a synchrotron source, but a lot can be achieved with state-of-the-art laboratory sources and detectors, and there is an obvious advantage with in-house measurements that the time constraints are less severe (although overlong measurements may land you in the LLC category, *i.e.* ‘Least Loved Coworker’).

It is also important because it shows how incommensurate structure analysis has graduated from a somewhat niche activity to one of the many crystallographic tools needed to study the full complexity of ordered solid-state systems.

## Figures and Tables

**Figure 1:**
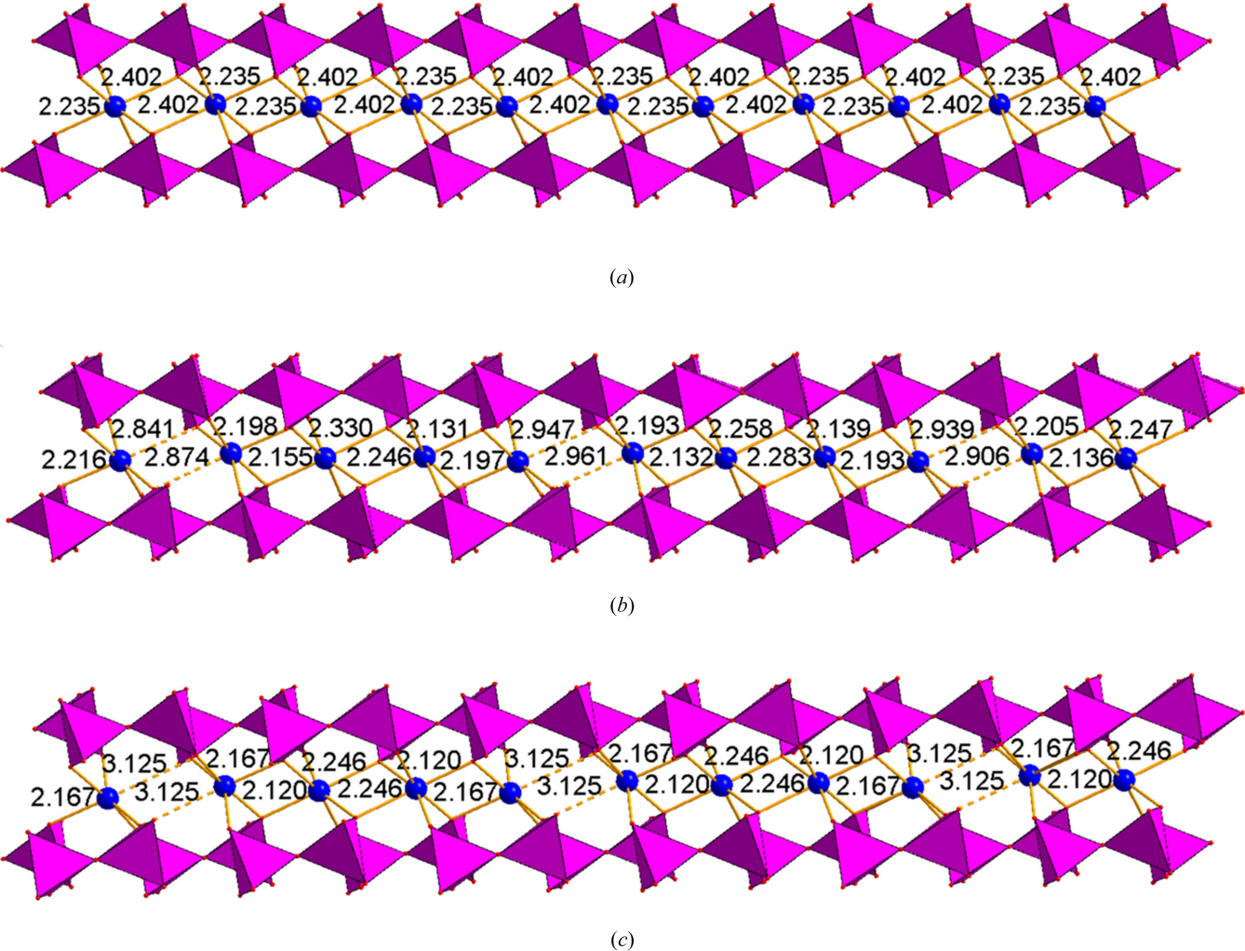
The three triclinic polymorphs of iron pyrophosphate. (*a*) α_3_, high-temperature triclinic polymorph; (*b*) α_2_, room-temperature triclinic polymorph, modulated structure; (*c*) α_1_, low-temperature triclinic polymorph. Note how the segregation between long and short Fe—O bonds gets progressively more pronounced as the temperature decreases. The concomitant deviation of the O—P—O angle from 180° that allows this degree of freedom is less obvious, but still discernible. Bonds longer than 2.5 Å have been highlighted by broken lines. While this limit is arbitrary, it is notable that in the α_2_ and α_1_ polymorphs there are no Fe—O bonds in the range 2.3–2.8 Å.
